# Glucose-montmorillonite hydrochar composite activating peroxymonosulfate for sulfentrazone rapid degradation and phytotoxicity alleviation to rice

**DOI:** 10.1007/s44297-024-00031-2

**Published:** 2024-07-05

**Authors:** Huan Yi, Guanghua Mo, Xuguo Zhou, Austin Merchant, Hailin Cai, Yaping Tao, Kailin Liu, Guolan Ma, Chunxia Ding, Xiangying Liu

**Affiliations:** 1 College of Plant Protection, Hunan Agricultural University, Changsha, 410128 China; 2 Institute of Plant Protection, Hunan Academy of Agricultural Sciences, Changsha, 410125 China; 3 Luo Ding Experiment Middle School, Yunfu, 527299 China; 4 Department of Entomology, School of Integrative Biology, College of Liberal Arts & Sciences, University of Illinois Urbana-Champaign, Urbana, IL 61801 USA; 5 Changsha Branch of Hunan Tobacco Company, Changsha, 410021 China; 6 College of Physics and Electronic Information & Henan Key Laboratory of Electromagnetic Transformation and Detection, Luoyang Normal University, Luoyang, 471934 China; 7 School of Chemistry and Materials Science, Hunan Agricultural University, Changsha, 410128 China; 8 Department of Entomology, Martin-Gatton College of Agriculture, Food and Environment, University of Kentucky, Lexington, KY 40546 USA

**Keywords:** Peroxymonosulfate, Advanced oxidation process, Sulfentrazone, Herbicide residue, Residue phytotoxicity

## Abstract

Sulfentrazone is an excellent herbicide for the control of broad-leaved weeds and some grasses. However, its herbicidal residues pose potential risks to the environment. The use of carbonaceous materials as catalysts for activating peroxymonosulfate (PMS) for managing pesticide contamination has attracted considerable attention. Herein, the rapid degradation of sulfentrazone in water and the alleviation of sulfentrazone-induced phytotoxicity alleviation to rice were investigated using glucose-montmorillonite hydrochar (GM-HC) activating PMS. The findings revealed that the GM-HC/PMS system can rapidly degrade sulfamethoxazole and achieved 93.90% of removal efficiency in 24 h. Specifically, GM-HC activating PMS generated the active species, including sulfate radicals (SO_4_•^–^), singlet oxygens (^1^O_2_) and superoxide radicals (O_2_•^−^), which speeds up the degradation of sulfentrazone. Five major degradation products of sulfentrazone and two possible degradation pathways were proposed. The C-Cl and C-CH_3_ of sulfentrazone molecules are the initiation sites attacked by active species for degradation according to density functional theory (DFT) calculation. Additionally, after the sulfentrazone was degraded by GM-HC/PMS system, sulfentrazone-induced phytotoxicity to rice seeds and seedlings was significantly alleviated. This work presents a novel approach for addressing herbicide wastewater pollution.

**Graphical Abstract:**

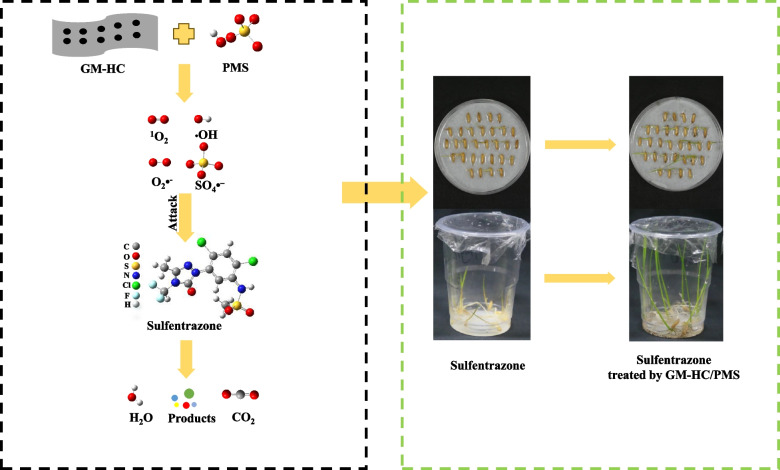

**Supplementary Information:**

The online version contains supplementary material available at 10.1007/s44297-024-00031-2.

## Introduction

Sulfentrazone, a protoporphyrinogen oxidase (PPO)-inhibiting herbicide, has been widely used for controlling broad-leaved weeds and some grasses (e.g., pigweed, waterhemp, and crabgrass) in soybean, sugarcane, and tobacco [1]. However, sulfentrazone is highly stable, and the degradation of sulfentrazone in the environment is very slow. Its half-life extension in soil could reach 541 d [2]. The solubility of sulfentrazone in water is 780.0 mg/L [3]. It can easily enter natural water bodies through rainfall and surface runoff leading to water contamination [4, 5]. It was documented that sulfentrazone has a high risk to aquatic plants. Kong et al. found that sulfentrazone is highly toxic to *Scenedesmus obliquus* with 72 h-ErC50 of 0.415 mg a.i./L and 72 h-EyC50 of 0.227 mg a.i./L, respectively [6]. Also, sulfentrazone can induce neurologic disorders in vertebrates and affect bacterial growth [7, 8]. Therefore, limiting sulfentrazone residue is crucial for minimizing its risks to the environment.

Traditionally, three major methods are used to reduce sulfentrazone contamination in water, including adsorption [9], electrochemical advanced oxidation [10] and microbial degradation [11]. Diogo et al. reported the Carbon nanostructures supported on Co/serpentinite is effective in adsorbing sulfentrazone in water [12]. However, most the porous adsorbents are unlikely to exhibit desirable adsorption capacity and desorption efficiency [9, 13]. Lima et al. adopted an electro-Fenton method for degrading sulfentrazone in water and achieved a mineralization of 60.0% for 200.0 mg/L sulfentrazone in 4 h [14]. However, the electrochemical degradation method may incur a high cost and toxic secondary metabolites [10]. Melo et al. isolated six bacterial strains for degrading sulfentrazone in water, but the degradation rate of sulfentrazone only ranged from 4.0% to 15.0% within 10 d [15]. Microbial degradation is mostly confined to the laboratory stage due to the poor survival ability of bacteria and fungi, as well as fluctuating residue degradation rates [16].

The activation of persulfate such as peroxymonosulfate (PMS) and persulfate (PS) using carbon-based materials for removing organic pollutants has been regarded as an efficient and friendly advanced oxidation processes (AOPs) in recent years [17, 18]. Compared to AOPs via semiconductor photocatalysis [19], sulfate radicals (SO_4_•^–^) are produced from activating persulfate in addition to hydroxide radicals (e.g., O_2_•^−^, ^1^O_2_ and •OH) generated by catalytic reactions [20]. SO_4_•^–^ possesses a longer half-life and stronger redox ability compared to hydroxide radicals, demonstrating more efficient oxidative activity in the degradation of organic pollutants [21, 22]. SO_4_•^–^ can easily oxidize pollutants containing unsaturated bonds or aromatic rings, resulting in partial or complete decomposition and mineralization into CO_2_, H_2_O, and inorganic ions [19]. In particular, AOPs based on SO_4_•^–^ have garnered increasing attention in recent years and have been used extensively in disinfection [23], environmental purification [24], and health care [25] due to their advantages of catalytic stability, high selectivity, high capability and adaptability, low cost, and environmentally friendly nature [17].

Generally, PS and PMS can be activated by heat, ultrasound, ultraviolet light, activated carbon, or transition metals to form SO_4_•^−^ [26]. Among these activation methods, carbon-based materials have been recognized as one of the most powerful approaches for PS and PMS activation due to their relatively high specific surface area, cost-effectiveness, environmentally friendly nature, high pore volume, and high catalytic activity [27]. Therefore, AOPs based on sulfate activated by carbon-based materials are also regarded as a promising new green technology for managing pesticide residues. Montmorillonite is a common clay mineral and has been widely used as an inorganic template for carbonous materials to enhance their dispersion and stability due to its large surface area and low cost [28]. It was documented that montmorillonite played important roles in the as-prepared carbon-based composites, which effectively enhanced the performance for activating PMS [29]. Among carbon-montmorillonite composites, hydrochar-montmorillonite has served as one of the most potential candidates for catalyzing PMS/PS with the advantages of simple preparation processes, low energy consumption, low cost, and high efficiency [17].

In this study, a glucose-montmorillonite hydrochar (GM-HC) composite was used to remove sulfentrazone residues by activating PMS in water. We (1) documented the effect of the main factors on the degradation efficiency of sulfentrazone, (2) determined the contribution of active species formed during the activation reaction for the rapid degradation of sulfentrazone, (3) identified the intermediates of sulfentrazone using PMS activated by GM-HM, proposed possible degradation pathways, and evaluated their toxicity, (4) predicted degradation products and the molecular mechanism of oxidation degradation of sulfentrazone by quantum chemistry analyses, and (5) investigated the alleviation of sulfentrazone-induced phytotoxicity to crops using the GM-HC/PMS system. This work is expected to provide a potential technique for the degradation of herbicide residues in wastewater.

## Materials and methods

### Materials

The GM-HC composite was prepared by our work team with a 1:1 mass ratio of glucose:natural montmorillonite. The preparation method and characterization of the GM-HC composite were described by Ding et al. First, 6.0 g of acid-treated montmorillonite and 6.0 g of glucose were added to 70 mL of deionized water and vigorously stirred for 4 h. Then, the mixture was transferred to a polytetrafluoroethylene-lined stainless steel autoclave and heated at 180 °C for 24 h. Finally, the prepared composite material was centrifuged, washed three times with distilled water and followed by twice with ethanol, and dried in an oven at 105 °C for 12 h [17]. Sulfentrazone with a minimum active substance purity of 95.0% was obtained from Luzhou Dongfang Agrochemical Co., Ltd. (Luzhou, China). PMS, NaOH, HCl, NaHCO_3_, NaCl, Na_2_SO_4_, NaNO_3_, NaH_2_PO_4_, 1,4-benzoquinone (p-BQ), tertbutyl alcohol (TBA), acetic acid and furfuryl alcohol (FFA) were of analytical grade and were purchased from Sinopharm Chemical Reagent Co., Ltd. (Shanghai, China). Methanol (MeOH) and acetonitrile were chromatographic grade and supplied by Tedia Company Inc. (Fairfield, USA). Seeds of rice of the Lingliangyou 942 variety were purchased from Longping Seed Company (Changsha, China).

### Sulfentrazone degradation

The sulfentrazone degradation procedure was as follows: first, 15.0 mg of GM-HC and 5.0 mg of PMS were added to 50.0 mL of 10.0 mg/L sulfentrazone, followed by shaking at 120 rpm at 25 °C. At set intervals, 1.0 mL of solution was collected, and the reaction was quenched immediately by adding 1.0 mL of MeOH. To determine the effect of various factors on sulfentrazone degradation by PMS activated with GM-HC, the initial concentration of sulfentrazone (2.50–40.0 mg/L), PMS dose (0.50–6.0 g/L), GM-HC amount (0.50–4.0 g/L), initial pH (3.35–8.51) adjusted using H_2_SO_4_ or NaOH solutions, reaction temperature (15–45 °C), and concentration of inorganic anions (Cl^−^, HCO_3_^−^, H_2_PO_4_^−^, NO_3_^−^, SO_4_^2−^) were evaluated. Three recycling runs of GM-HC were carried out to evaluate its reusability under the same reaction conditions. The concentration of remaining sulfentrazone was determined by high-performance liquid chromatography (HPLC), and the degradation intermediates and products of sulfentrazone were detected by UPLC-QTOF/MS after filtration with a 0.22 µm filter.

### Analysis of active species

To identify the active species generated during the GM-HC activation of PMS, we conducted quenching experiments using various scavengers. MeOH at 10.0, 50.0, or 100.0 mM was used to quench SO_4_•^–^ and •OH; TBA at 10.0, 50.0, or 100.0 mM was used for •OH; p-BQ at 5.0, 10.0, or 20.0 mM was used for O_2_•^−^, and FFA at 5.0, 10.0, or 20.0 mM was used for ^1^O_2_ [30]. A certain amount of scavenger was added to a 100.0 mL glass beaker containing 10.0 mg/L sulfentrazone, 3.0 g/L GM-HC and 1.0 g/L PMS by shaking at 120 rpm at 25 °C. At 0, 1, 8, 12, 16, and 24 h, 1.0 mL of solution was added to 1.0 mL of MeOH. The sample underwent filtration using a 0.22 µm filter, and the amount of sulfentrazone was measured using HPLC.

### HPLC and UPLC-QTOF-MS analysis

The amount of sulfentrazone remaining following degradation was analyzed using an HPLC system (Agilent LC1260, USA) with a Kromasil® C18 column (250.0 mm × 4.60 mm, 5.0 µm). Sulfentrazone was detected by an ultraviolet detector at 240.0 nm with a mobile phase (MeOH:water containing 0.10% acetic acid), 70:30, v/v) at a flow rate of 0.80 mL/min. The degradation products of sulfentrazone were analyzed by UPLC-QTOF-MS (Agilent UPLC1290-QTOF6530, USA) by ESI negative ion injection voltage with an ACCHROM XAqua C18 column (150 mm × 4.6 mm, 5 μm) under the following conditions: mobile phase (0.20% acetic acid solution in water and acetonitrile) with a flow rate of 0.30 mL/min and a column temperature of 35 °C. The scanning range (m/z) was 50–500 amu.

### DFT calculations

DFT calculations were used to predict the reactive sites for nucleophilic, electrophilic, and radical attacks based on Fukui functions [31]. The geometric conformation of the sulfentrazone molecule was established using GaussView 5.0.8. Then, geometric optimization and frequency analysis were performed using Gaussian 09 at the B3LYP/6-31G* level. Frontier electron density (FED) describes the electronic properties and reactivity of molecules and predicts the contribution rates of the highest occupied molecular orbital (HOMO) and the lowest unoccupied molecular orbital (LUMO) to identifying reaction sites in molecules [32]. The frontier electron density of the HOMO (${FED}_{HOMO}^{2}$) and the frontier electron density of the LUMO (${FED}_{LUMO}^{2}$) were calculated using the method described by Lee et al. [33].

### Detoxification bioassay

To investigate the toxicity caused by sulfentrazone and detoxification of sulfentrazone in rice by GM-HC/PMS, rice was selected as a target for toxicity assessment because it is very sensitive to sulfentrazone. Bioassays were conducted in Petri dishes for rice seed germination and in plastic cups for rice growth. All dishes and cups were put into a growth chamber at 25/28 °C night/d under a 10 h-dark and 14 h-light cycle with a 12,000 lx light intensity and 80.0% relative humidity. For the seed germination bioassays, a total of 30 seeds were evenly placed in a 9-cm diameter Petri dish. Two pieces of filter paper were placed in a dish and moistened with 5.0 mL of one of three aqueous solutions (distilled water, solution with concentrations of 0.20, 1.0, and 5.0 mg/L of sulfentrazone, or solution of sulfentrazone treated with 3.0 g/L of GM-HC coupled 1.0 g/L of PMS). Germination was monitored for 5 d and the percentage of seeds that germinated seeds was recorded (Eq. (1)). For the rice growth bioassays, a total of 10 germinated rice seeds were exposed to 5 mL of one of the three aqueous solutions described above. After 5 d of growth, the length of the rice shoots was measured.

1 $$\mathrm{Germination}\;\mathrm{rate}\;(\%)\;=\;(\mathrm{germinated}\;\mathrm{seeds}/\;\mathrm{total}\;\mathrm{number}\;\mathrm{of}\;\mathrm{seeds})\;\times\;100$$

### Toxicity analysis

The toxicity of sulfentrazone and its intermediates was predicted by the Toxicity Evaluation Software Tool (T.E.S.T., 19.0.0.0) program.

### Data analysis

The data were presented as means ± standard deviations of three replicates. The significance of differences between the treatments was assessed using an independent-samples t-test, *P < 0.05.

## Results and discussion

### Effect of PMS concentration on sulfentrazone removal

**Fig. 1:**
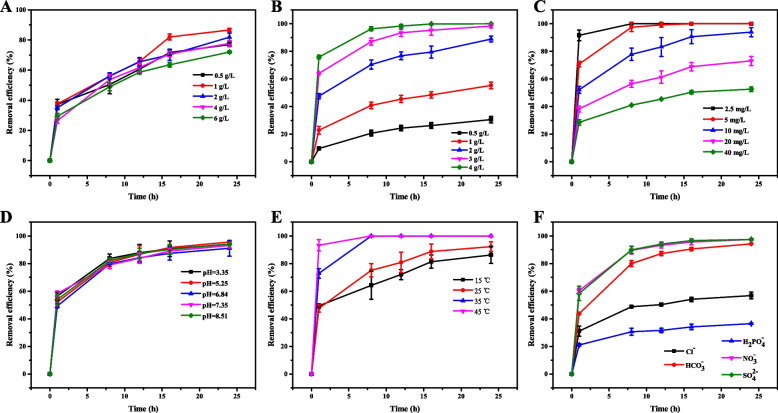
Effect of main experimental factors on sulfentrazone removal efficiency. **A** PMS concentration; (**B**) GM-HC dosage; (**C**) Initial concentration of sulfentrazone; (**D**) Initial solution pH; (**E**) Temperature; (**F**) Common anions

The effect of the PMS concentration on sulfentrazone removal was studied over a range of 0.50–6.0 g/L. The 24 h sulfentrazone removal efficiency increased from 76.30% to 86.30% as the PMS concentration increased from 0.50 g/L to 1.0 g/L (Fig. 1A). However, with further increase of the concentration of PMS to 6.0 g/L, the removal efficiency was reduced to 72.0%. Enhancing PMS concentration generates more sulfate radicals, leading to an increase in the sulfentrazone removal efficiency [34, 35]. However, SO_4_•^−^ and •OH may also be scavenged through undesirable reactions when the PMS concentration is high, resulting in a decrease in sulfentrazone removal efficiency that we observed at concentrations > 1.0 g/L (Eqs. (2) and (3)) [36, 37].

2 $$\bullet \text{OH }+\text{ HS}\mathrm{O}_{5}^{-}\to \mathrm{SO}_{5}\bullet^{-} + {\mathrm{H}}_{2}\mathrm{O}$$

3 $$\mathrm{SO}_4\bullet^-+\text{ HS}{{\mathrm{O}}_{5}}^{-}\rightarrow\text{ S}\mathrm{O}_5\bullet^-+\text{ HS}{{\mathrm{O}}_{4}}^{-}$$

### Effect of the dose of GM-HC on sulfentrazone removal

Sulfentrazone removal was measured at diverse GM-HC dosages. The GM-HC dose significantly affected sulfentrazone removal (Fig. 1B). The removal efficiency of sulfentrazone was enhanced when the GM-HC dose was increased from 0.50 g/L to 4.0 g/L. Higher amounts of GM-HC resulted in higher removal efficiency because of an increase in the number of active sites available for PMS decomposition to produce more active species (e.g., SO_4_•^−^, O_2_•^−^, and •OH) [17, 38, 39]. When the GM-HC dose was increased from 3.0 g/L to 4.0 g/L, the removal efficiency of sulfentrazone reached 100.0% during a 24-h reaction with the GM-HC/PMS system. It was reported that an excessive amount of catalyst can lead to aggregation, hindering the reaction between pollutants and free radicals [40]. However, Ding et al. found that increasing the concentration of hydrochar-montmorillonite from 0.60 g/L to 3.0 g/L could catalyze PMS to produce more reactive species, thereby enhancing the removal efficiency of dicamba [17], which is consistent with our empirical data in this study.

### Effect of initial sulfentrazone concentration on removal

The initial concentration of sulfentrazone significantly affected its removal efficiency (Fig. 1C). The removal efficiency of sulfentrazone decreased as its concentration increased. After 24 h, the removal efficiencies of sulfentrazone at concentrations of 2.5, 5.0, 10.0, 20.0, and 40.0 mg/L were 100.0%, 100.0%, 93.90%, 73.0%, and 52.60%, respectively. Because the PMS dose remained fixed as the sulfentrazone concentration increased, this fixed PMS dose was not sufficient to completely remove sulfentrazone from the system at higher concentrations [41]. Ma et al. observed that the removal efficiency of imidacloprid was negatively correlated with its initial concentration in the G-HWTRs/PMS system [42], and Ding et al. reported similar results for quinclorac removal in the nZVI/ATP-PMS system [43].

### Effect of initial pH on sulfentrazone removal

The removal efficiencies of sulfentrazone were 95.61%, 95.51%, 94.20%, 94.10%, and 93.10% after 24 h of reaction at initial pH values of 3.35 (unadjusted pH), 5.25, 6.84, 7.35, and 8.51, respectively (Fig. 1D). This result indicates that the removal efficiency of sulfentrazone is minimally influenced by the pH levels in acidic, neutral, and alkaline conditions when employing the GM-HC/PMS system, enabling effective operation across a broad pH range [44]. These findings are in line with those reported by Qi et al. [45].

### Effect of temperature on sulfentrazone removal

Sulfentrazone removal efficiency was positively correlated with temperature in the GM-HC/PMS system (Fig. 1E). 8 h sulfentrazone removal efficiency increased from 64.25% to 100.0% when the reaction temperature was elevated from 15 °C to 45 °C. Temperature is a crucial factor in the activation of PMS. Li et al. found that the efficiency of ofloxacin degradation by heat-activated persulfate was dramatically enhanced with increased temperature [46]. Ding et al. also demonstrated that the degradation of quinclorac was significantly accelerated with increasing temperature in the presence of nZVI/ATP3 activating PMS [43]. Thus, temperature could contribute to the diffusion rate of PMS and accelerate generation of SO_4_•^–^ and •OH, enhancing removal efficiency [47].

### Effects of anions on sulfentrazone removal

The addition of anions significantly influenced the sulfentrazone removal efficiency (Fig. 1F). The removal efficiencies of sulfentrazone were 56.85%, 94.25%, 34.05%, 97.21% and 97.29% after 24 h of reaction involving Cl^−^, HCO_3_^−^, H_2_PO_4_^−^, NO_3_^−^, and SO_4_^2−^, respectively, and were 93.32% without the addition of anions. This result indicates that the presence of Cl^−^ and H_2_PO_4_^−^ decreased the removal efficiency of sulfentrazone, perhaps because the generated SO_4_•^−^ and •OH could react with Cl^−^, as demonstrated in Eqs. (4)-(8), and H_2_PO_4_^−^, as demonstrated in Eq. (8), to yield fewer reactive radicals [34, 48, 49]. The unexpected phenomenon of the removal efficiency of sulfentrazone lower by adding H_2_PO_4_^−^ reaction than by adding Cl^−^ might be due to the reason that excess Cl^−^ reacted with PMS and Cl• to produce reactive halogens such as Cl_2_•^−^ and HOCl, which also could oxidize sulfentrazone according to Eqs. (4)-(8). In contrast, HCO_3_^−^, NO_3_^−^ and SO_4_^2−^ had little effect on the removal efficiency of sulfentrazone by the GM-HC/PMS system [50].

4 $$\mathrm{HS}{{\mathrm{O}}_{5}}^{-}+{\mathrm{Cl}}^{-}\to \mathrm{S}{{\mathrm{O}}_{4}}^{2-}+\mathrm{HOCl}$$

5 $$\mathrm{HS}{{\mathrm{O}}_{5}}^{-}+2{\mathrm{Cl}}^{-}+{\mathrm{H}}^{+}\to \mathrm{S}{{\mathrm{O}}_{4}}^{2-}+{\mathrm{Cl}}_{2}+{\mathrm{H}}_{2}\mathrm{O}$$

6 $$\mathrm{SO}_{4}\bullet^{-}+\mathrm{Cl}^{-}\to \mathrm{Cl}\bullet+\mathrm{S}{{\mathrm{O}}_{4}}^{2-}$$

7 $$\mathrm{Cl}\bullet+\text{ C}{\mathrm{l}}^{-}\to \mathrm{Cl}_{2}\bullet^{-}$$

8 $$\bullet \mathrm{OH}+\mathrm{H}_{2} \mathrm{PO}_{4}^{-}\to \mathrm{OH}^{-}+\mathrm{H}_{2} {\text {PO}}_{4} \bullet$$

### Reusability of GM-HC

**Fig. 2:**
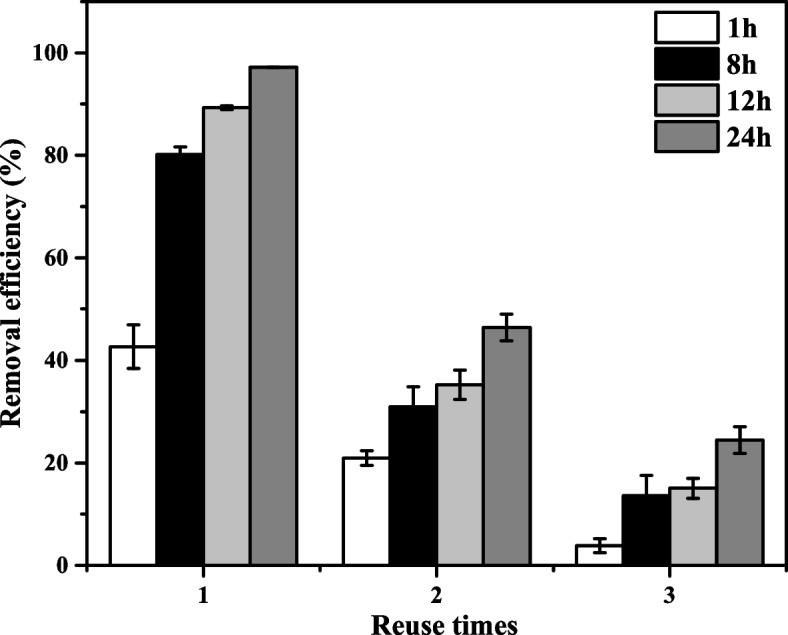
Effect of the reuse times of GM-HC on sulfentrazone removal efficiency

To assess the reusability of GM-HC activating PMS for removing sulfentrazone, sulfentrazone degradation was carried out three successive cycles at the same reaction condition. Figure 2 showed the sulfentrazone removals for three consecutive runs. As observed, the degradation efficiency of sulfentrazone gradually decreased with increasing reuse time. The degradation efficiency of sulfentrazone decreased from 97.16% to 24.44% after the third cycle. A possible reason for this decrease is the deposition of degradation products on the surface of the GM-HC, leading to a decrease in the number of available reaction sites for activating PMS [51].

### Determination of active species

**Fig. 3:**
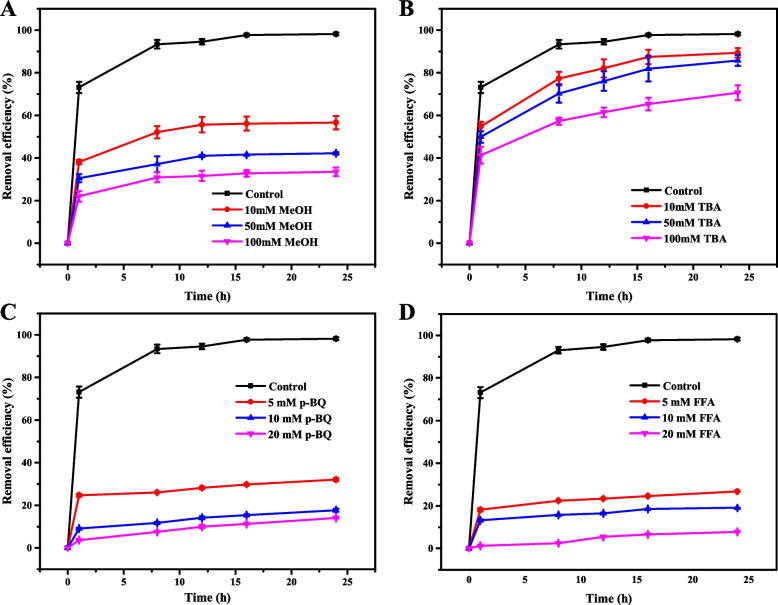
Effects of scavengers on sulfentrazone removal. **A** Methanol (MeOH); (**B**) Tertbutyl alcohol (TBA); (**C**) 1,4-benzoquinone (p-BQ); (D) Furfuryl alcohol (FFA)

The active species generated during sulfentrazone removal in the GM-HC/PMS system were identified using several scavengers [52]. The results showed that the four scavengers MeOH, TBA, p-BQ, and FFA had adverse effects on the sulfentrazone removal efficiency in the GM-HC/PMS system, and this inhibition of sulfentrazone removal became more pronounced as the scavenger concentration increased (Fig. 3). Sulfentrazone removal efficiency ranged from 32.83% to 57.96% when MeOH was added to the reaction system, indicating that SO_4_•^−^ and •OH play crucial roles in the degradation of sulfentrazone (Fig. 3A). Sulfentrazone removal efficiency ranged from 70.61% to 87.68% when TBA was used to quench •OH, suggesting that SO_4_•^−^ contributes more to sulfentrazone removal than •OH (Fig. 3B). Both p-BQ and FFA had significant inhibitory effects on sulfentrazone removal efficiency, reducing the removal efficiency to below 25.0%, suggesting that large amounts of O_2_•^−^ and ^1^O_2_ were involved in these reactions (Fig. 3C, D). These combined results suggest that SO_4_•^−^, O_2_•^−^, and ^1^O_2_ are the dominant active species in sulfentrazone degradation. These species may be attributed to the oxygen-containing functional groups, including doublet aliphatic C-OH and C = O and phenolic C-O/Si-O, on the surface of GM-HC transferring electrons to PMS, causing the cleavage of the peroxide O-O bond in PMS to form SO_4_•^−^, which further generates O_2_•^−^ and ^1^O_2_. The reaction of glucose-montmorillonite hydrochar composite activating PMS facilitates sulfentrazone degradation [17].

### Degradation products and pathway of sulfentrazone

The degradation intermediates and products were detected by UPLC-QTOF/MS. The mass spectrum of sulfentrazone and its degradation intermediates were displayed in Supplementary Materials Fig. S1A-F. Sulfentrazone (C_11_H_10_Cl_2_F_2_N_4_O_3_S,* m*/*z* 384.98) and its five degradation intermediates were identified as 1-(2,4-dichloro-5-(methylsulfonamido)phenyl)-4-(difluoromethyl)-5-oxo-4,5-dihydro-1-H-1,2,4-triaz-ole-3-carboxylic acid (S1,C_11_H_8_Cl_2_F_2_N_4_O_5_S,* m*/*z* 414.82), N-(2,4-dichloro-5-(4-(difluoromethyl)-3-(hydroxyl-methyl)-5-oxo-1,2,4-triazolidin-1-yl)phenyl)methanesulfonamide (S2,C_11_H_12_Cl_2_F_2_N_4_O_4_S, *m*/*z* 402.80), N-(4-chloro-3-(4-(difluoromethyl)-3-methyl-5-oxo-4,5-dihydro-1H-1,2,4-triazol-1-yl)phenyl)methanesulfonamide (S3,C_10_H_8_C_l2_F_2_N_4_O_3_S, *m*/*z* 370.81), N-(3-(3,4-dimethyl-5-oxo-4,5-dih-ydro-1H-1,2,4-triazol-1-yl)phenyl) methanesulfonamide (S4, C_11_H_11_ClF_2_N_4_O_3_S, *m*/*z* 351.02), and 4-(difluorom-ethyl)-5-methyl-2,4-dihydro-3H-1,2,4-triazol-3-one (S5, C_11_H_14_N_4_O_3_S,* m*/*z* 281.04) (Supplementary Materials Table S1).

**Fig. 4:**
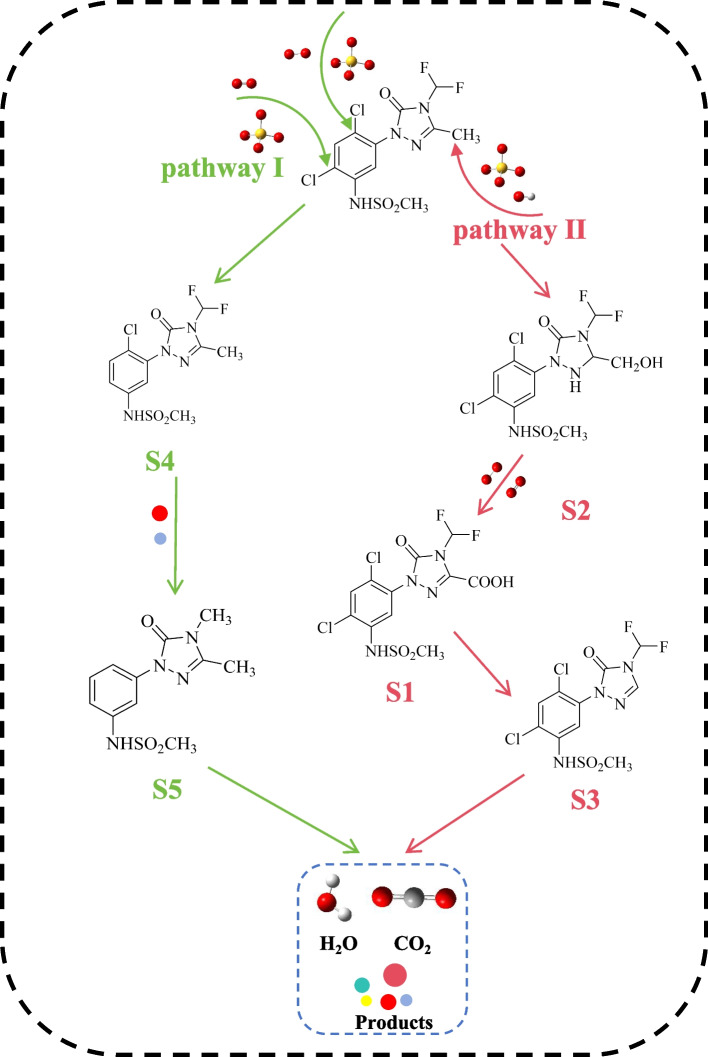
Proposed degradation pathways of sulfentrazone in GM-HC/PMS system

Based on the chemical structure of sulfentrazone and its metabolites, two potential degradation pathways of sulfentrazone in the GM-HC/PMS system have been suggested (Fig. 4). In the first proposed pathway, the chlorine atoms on the benzene ring of the sulfentrazone molecule were removed by attacking •OH, leading to the generation of intermediate S4 followed by S5. The dechlorination of hydrogen atoms substituting for chlorine atoms has been documented in the degradation pathway of quinclorac [53]. After the dechlorination of sulfentrazone, S5 is oxidized by the active species into small inorganic molecules, CO_2_, and H_2_O. In the second proposed pathway, a hydrogen atom on the methyl group (-CH_3_) in the triazol ring is initially removed under the energy of pπ-conjugation to form a methylene radical (-CH_2_•), which binds with •OH to produce hydroxymethyl (-CH_2_OH), leading to the generation of the metabolite S2. Then, the -CH_2_OH group at the triazol ring of sulfentrazone undergoes oxidation to a carboxyl group (–COOH) upon attack by SO_4_•^−^, ^1^O_2_, and O_2_•^−^, leading to the formation of product S1 [54, 55]. Subsequently, a decarboxylation reaction occurs to produce S3 [17, 56]. Considering these two pathways, it is highly plausible that sulfentrazone undergoes stepwise degradation involving substitution, oxidation, decarboxylation, and dechlorination, ultimately mineralizing into CO_2_, H_2_O, and small inorganic molecules.

### Quantum chemistry analysis of sulfentrazone degradation

**Fig. 5:**
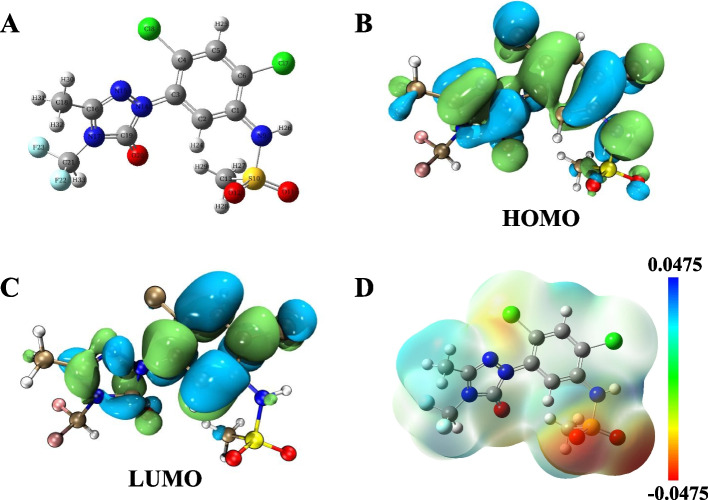
DFT calculations on sulfentrazone molecule. **A** Optimized chemical structure of sulfentrazone; (**B**) The highest occupied molecular orbital (HOMO); (**C**) The lowest unoccupied molecular orbital (LUMO); (**D**) Electrostatic potential (ESP)-mapped molecular surface of sulfentrazone

To further demonstrate the degradation products obtained from UPLC-QTOF/MS and the degradation pathway of sulfentrazone, quantum chemical analyses of sulfentrazone were performed based on the optimal geometric conformation of the sulfentrazone molecule obtained at the B3LYP/6-31G* level. The spatial configuration of the sulfentrazone molecule is presented in Fig. 5A. As an effective approach for predicting the degradation products and pathways of organic compounds, DFT calculations based on the HOMO, LUMO, and electrostatic potential (ESP) surface, ${\mathrm{FED}}_{\mathrm{HOMO}}^{2}$ and ${\mathrm{FED}}_{\mathrm{LUMO}}^{2}$ have been broadly employed to explain the reactivity sites on organic molecules [24, 54]. The HOMO represented the most electron-rich regions at C 4, C 6, Cl 7, C1 8, and N 17, and the LUMO represented electron-deficient sites at C 4, C 6, C1 8, and N 17 (Fig. 5B, C). Moreover, the FED values of the HOMO and LUMO indicate that C 4 and C 6 possess the highest FED_HOMO_^2^ and FED_LUMO_^2^, with values of 0.2506 and 0.3650, respectively (Table 1).

**Table 1:** Frontier electron density of highest occupied molecular orbital (HOMO) and lowest unoccupied molecular orbital (LUMO) on major atoms of sulfentrazone at the B3LYP/6-31G* level

Atom	FED^2^_LUMO_	FED^2^_HOMO_	Atom	FED^2^_LUMO_	FED^2^_HOMO_
1(C)	0.1100	0.1768	18(C)	0.0073	0.0125
2(C)	0.2970	0.0487	19(C)	0.0632	0.0465
3(C)	0.3642	0.1373	20(O)	0.0357	0.1256
4(C)	0.1123	0.2506	21(C)	0.0020	0.0018
5(C)	0.2584	0.0536	22(F)	0.0010	0.0004
6(C)	0.3650	0.2062	23(F)	0.0003	0.0011
7(Cl)	0.0952	0.1057	24(H)	0.0300	0.0028
8(Cl)	0.0169	0.1798	25(H)	0.0271	0.0022
9(N)	0.0112	0.2129	26(H)	0.0015	0.0159
10(S)	0.0056	0.0336	27(H)	0.0006	0.0012
11(O)	0.0007	0.0164	28(H)	0.0012	0.0028
12(O)	0.0009	0.0046	29(H)	0.0085	0.0018
13(C)	0.0078	0.0119	30(H)	0.0005	0.0010
14(N)	0.0607	0.1548	31(H)	0.0046	0.0068
15(N)	0.0504	0.0788	32(H)	0.0019	0.0053
16(C)	0.0428	0.0802	33(H)	0.0003	0.0003
17(N)	0.0153	0.0198	-	-	-

Figure 5 showed that atoms C 4 and C 6 represent vulnerable sites attacked by radicals during sulfentrazone degradation, leading to cleavage of C 6-Cl 7 and C 4-Cl 8 and the generation of degradation products S4 and S5. This theoretical research on frontier electron densities was reported by Wang et al. to elucidate the degradation of indomethacin [57]. Figure 5D shows the ESP, highlighting the susceptibility of C 4, Cl 7, Cl 8, N 17, C 18, and O 20 to attack by electrophilic free radicals. These sites are easier for active species to attack and exhibit high degradation activity [30, 58]. The quantum analyses aligned with the degradation products identified through UPLC-QTOF/MS and the suggested degradation pathway. These findings provide valuable insights into the reactivity of sulfentrazone, aiding in a comprehensive understanding of its degradation behavior in the GM-HC/PMS system.

### Detoxification of sulfentrazone by GM-HC/PMS

**Fig. 6:**
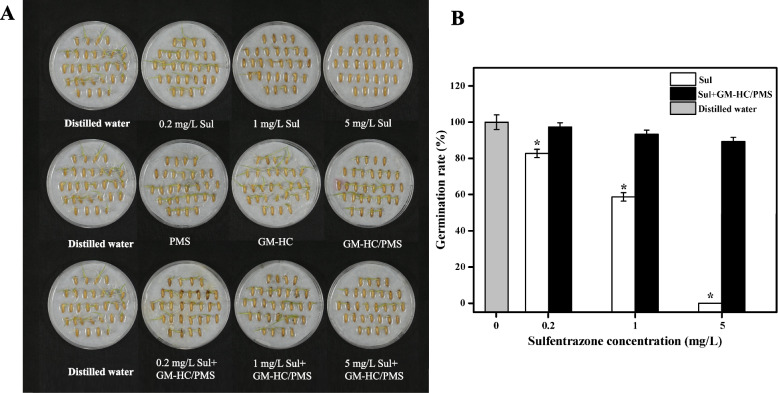
Rate of rice seed germination in sulfentrazone solution with treatment by the GM-HC/PMS system after 5 days growth. Note: Sul: sulfentrazone. Asterisks indicate significant differences: *, *P* < 0.05, 0.01 by Student’s t-test

**Fig. 7:**
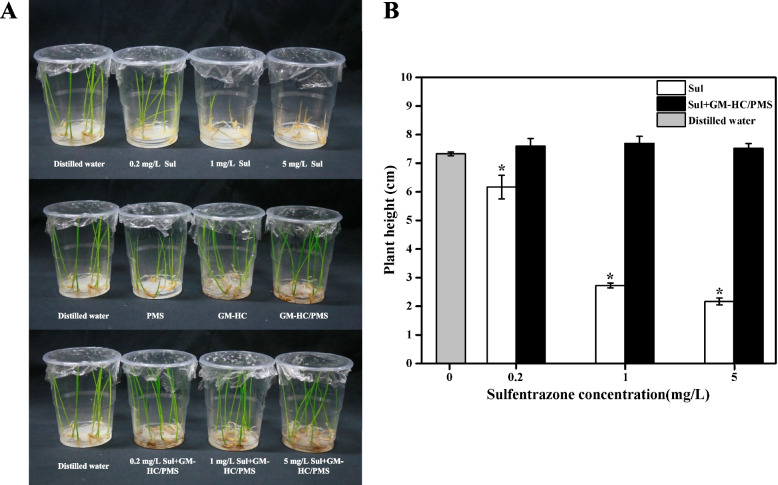
Effect of sulfentrazone with/without GM-HC/PMS treatment on shoot length of rice seedlings. Note: Sul: sulfentrazone. Asterisks indicate significant differences: *, *P* < 0.05, 0.01 by Student’s t-test

Previous studies have shown that sulfentrazone can cause serious phytotoxicity to sensitive crops [59, 60]. To evaluate the detoxification effect of the GM-HC/PMS system against sulfentrazone-induced phytotoxicity to rice, rice germination and seedling growth were investigated. The results showed that sulfentrazone significantly suppressed rice seed germination (Fig. 6A) and seedling growth (Fig. 7A) due to herbicidal phytotoxicity. The degree to which phytotoxicity was alleviated by the GM-HC/PMS treatment varied similarly between the rice germination and seedling growth groups. Under exposure to sulfentrazone at 0.20, 1.0, and 5.0 mg/L, the percentage of germinated seeds increased by 14.67%, 34.67%, and 89.33%, respectively, in the GM-HC/PMS treatment group compared to that in the untreated control group (Fig. 6B), and the shoot length increased by 19.42%, 67.86%, and 73.05%, respectively (Fig. 7B). The rice seed germination rate and seedling height were not significantly different in GM-HC/PMS-treated plants relative to those kept in distilled water. Moreover, it was also found that both 3.0 g/L GM-HC and 1.0 g/L PMS were benificial to plant growth. This phenomenon of good to crop growth was documented in previous research. Ding et al. observed that 3.0 g/L of hydrochar-montmorillonite (HC-Mt) and 0.61 g/L of PMS promoted the growth of mung bean [17]. Wen et al. utilized 12.0 g/L of biochar (WCGBC800) activating 60.0 g/L of PMS to alleviate the inhibitory effects of quinclorac on rice. They discovered that WCGBC800 and PMS promoting seed germination and seedling growth [61]. There was few reports on the overuse of catalysts and PMS affecting plant growth.

To comprehensively assess the ecological risks of the GM-HC/PMS system, the ecological toxicity of sulfentrazone and its degradation intermediates was predicted based on the Toxicity Estimation Software Tool (T.E.S.T.) [53]. The results are shown in Table 2. The results revealed that the toxicity of the degradation intermediates to Daphnia magna and the fathead minnow was lower than that of sulfentrazone, indicating that the GM-HC/PMS system can detoxify sulfentrazone to aquatic organisms, which is consistent with the alleviation of sulfentrazone-induced phytotoxicity to rice. Regarding the remediation efficiency of existing methods for sulfentrazone degradation and phytotoxicity alleviation in rice, the adsorption of biochar and multi-walled carbon nanotubes was able to alleviate the phytotoxicity of sulfentrazone to rice [62], but residual sulfentrazone remains in the environment after desorption. Similarly, Co^2+^ and Fe^2+^/PMS System were also used to alleviate the phytotoxicity of sulfentrazone to rice, but metal ions leaching causes secondary environmental pollution [63]. These combined results illustrate that the GM-HC/PMS system is a viable alternative for sulfentrazone detoxification.

**Table 2:** The toxicity of sulfentrazone and its degradation intermediates to Daphnia magna and Fathead minnow calculated by T.E.S.T software program

Compound	Chemical Formula	Fathead minnow LC_50_-96 h(mg/L)	Daphnia magna LC_50_-48 h(mg/L)
Sulfentrazone	C_11_H_10_Cl_2_F_2_N_4_O_3_S	0.65	12.73
S1	C_11_H_8_Cl_2_F_2_N_4_O_5_S	0.50	49.48
S2	C_11_H_12_Cl_2_F_2_N_4_O_4_S	3.11	60.54
S3	C_10_H_8_C_l2_F_2_N_4_O_3_S	1.74	14.08
S4	C_11_H_11_ClF_2_N_4_O_3_S	3.72	25.76
S5	C_11_H_14_N_4_O_3_S	29.20	35.09

## Conclusions

GM-HC catalyzes PMS to accelerate the degradation of sulfentrazone in water. When GM-HC was 3.0 g/L and PMS was 1.0 g/L, a removal efficiency of 10.0 mg/L for sulfentrazone reached 93.90% within 24 h. The main reason is that the SO_4_•^−^, O_2_•^−^, and ^1^O_2_ generated by GM-HC/PMS contributed to rapid degradation. Five degradation intermediates of sulfentrazone were detected, and two pathways were proposed, which were confirmed by DFT calculations. In addition, a phytotoxicity bioassay verified that GM-HC/PMS could alleviate the effects of sulfentrazone toxicity in rice seeds and seedlings. Our combined results indicate that GM-HC, which activates PMS, is a feasible alternative for addressing pesticide residues and their phytotoxicity to crops.

## Supplementary Information

Supplementary Material 1.

## Data Availability

Data will be made available upon reasonable request.
