# Supplementary material for: Glucose-montmorillonite hydrochar composite activating peroxymonosulfate for sulfentrazone rapid degradation and phytotoxicity alleviation to rice
Source: Crop Health. 2024 Jul 5;2(1):10. doi: 10.1007/s44297-024-00031-2 (PMC12825995; doi:10.1007/s44297-024-00031-2)
Supplement: Supplementary file 1 — Supplementary Material 1. [file 44297_2024_31_MOESM1_ESM.docx]

# Supplementary Materials

**Glucose-montmorillonite hydrochar composite activating peroxymonosulfate for sulfentrazone rapid degradation and phytotoxicity alleviation to rice**

Huan Yi ^1,^ †, Guanghua Mo ^1,3,^ †, Xuguo Zhou ^4^, Austin Merchant ^8^, Hailin Cai ^5^, Yaping Tao ^6^, Kailin Liu ^1^, Guolan Ma^2,^ *, Chunxia Ding^1,7,^ *, Xiangying Liu ^1,^*

*^1^ College of Plant Protection, Hunan Agricultural University, Changsha, 410128, China;*

*^2^ Hunan Provincial Key Laboratory for Biology and Control of Weeds, Hunan Academy of Agricultural Sciences, Changsha, 410125, China;*

*^3^ Luo Ding Experiment Middle School, Yunfu, 527299, China;*

*^4^ Department of Entomology, School of Integrative Biology, College of Liberal Arts & Sciences University of Illinois Urbana-Champaign, Urbana, IL, 61801, USA;*

*^5^ Changsha Branch of Hunan Tobacco Company, Changsha, 410021, China;*

*^6^ College of Physics and Electronic Information & Henan Key Laboratory of Electromagnetic Transformation and Detection, Luoyang Normal University, Luoyang, 471934, China;*

*^7^ School of Chemistry and Materials Science, Hunan Agricultural University, Changsha, 410128, China;*

*^8^ Department of Entomology, Martin-Gatton College of Agriculture, Food and Environment, University of Kentucky, Lexington, KY, 40546, USA;*

**Supplementary Table S1 The** **structural formula, chemical formula and m/z of sulfentrazone and its intermediates**

| Compound | Structural formula | Chemical formula | *m/z* |
| --- | --- | --- | --- |
| Sulfentrazone |  | C_11_H_10_Cl_2_F_2_N_4_O_3_S | 384.98 |
| S1 |  | C_11_H_8_Cl_2_F_2_N_4_O_5_S | 414.82 |
| S2 |  | C_11_H_12_Cl_2_F_2_N_4_O_4_S | 402.80 |
| S3 |  | C_10_H_8_C_l2_F_2_N_4_O_3_S | 370.81 |
| S4 |  | C_11_H_11_ClF_2_N_4_O_3_S | 351.02 |
| S5 |  | C_11_H_14_N_4_O_3_S | 281.04 |


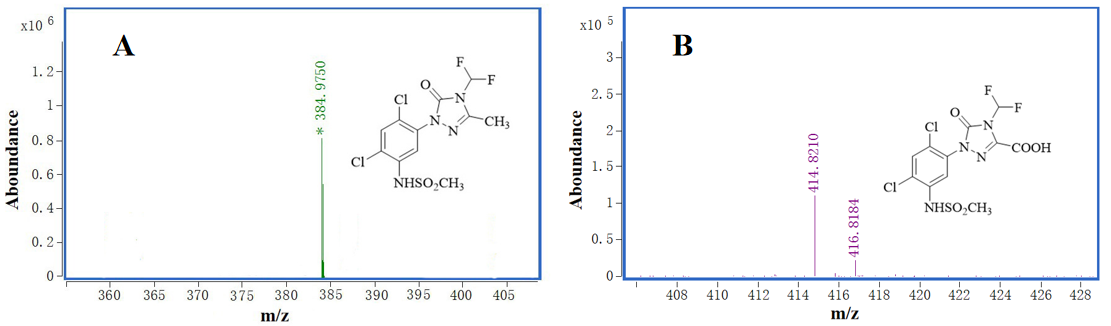


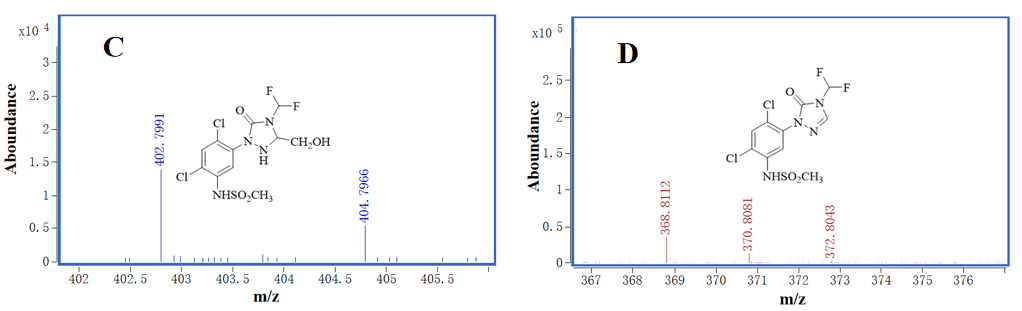

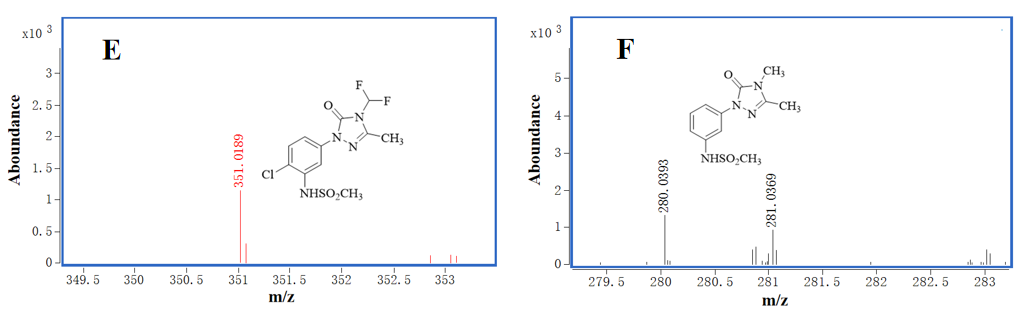


**Supplementary Fig. S1 The mass spectrum of sulfentrazone and degradation intermediates**.

A, sulfentrazone; B, Product S1 1-(2,4-dichloro-5-(methylsulfonamido)phenyl)-4-(difluoromethyl)-5-oxo-4,5-dihydro-1-H-1,2,4-triaz-ole-3-carboxylic acid; C, Product S2 N-(2,4-dichloro-5-(4-(difluoromethyl)-3-(hydroxyl-methyl)-5-oxo-1,2,4-triazolidin-1-yl)phenyl)methanesulfonamide; D, Product S3 N-(4-chloro-3-(4-(difluoromethyl)-3-methyl-5-oxo-4,5-dihydro-1H-1,2,4-triazol-1-yl)phenyl)methanesulfonamide; E, Product S4 N-(3-(3,4-dimethyl-5-oxo-4,5-dih-ydro-1H-1,2,4-triazol-1-yl)phenyl) methanesulfonamide; F, Product S5 4-(difluorom-ethyl)-5-methyl-2,4-dihydro-3H-1,2,4-triazol-3-one.
